# The prevalence and genetic disorders spectrum of thalassemia among breast cancer patients in Jiangxi province, China

**DOI:** 10.3389/fgene.2022.1001369

**Published:** 2022-10-18

**Authors:** Jingxian Ding, Zhaohui Huang, Xiaoliu Jiang, Qingge Li, Yali Cao, Yonghong Guo

**Affiliations:** ^1^ Department of Radiation Oncology, The Breast Cancer Institute, The Third Hospital of Nanchang, Nanchang, China; ^2^ Department of Breast Surgery, The Breast Cancer Institute, The Third Hospital of Nanchang, Nanchang, China; ^3^ Department of Radiation Oncology, The Fourth Affiliated Hospital of Nanchang University, Nanchang, China

**Keywords:** anemia, thalassemia, breast cancer, serum ferritin, molecular mutation spectrum

## Abstract

**Background:** Thalassemia is a common inherited hematological disease with genetic disorders characterized by imbalanced synthesis of the globin chains. Due to the improvement of treatment methods, patients with thalassemia can survive for a long time. Therefore, it is not uncommon for patients with thalassemia suffering from malignant tumors. However, there are quite few reports on thalassemia patients complicated with breast cancer. Herein, we try to investigate the prevalence and genetic disorders spectrum of thalassemia in patients with breast cancer.

**Methods:** Blood routing tests and serum ferritin analysis were conducted in 1887 breast cancer patients treated in the department of radiation oncology during 1 April 2020 and 30 March 2022. The suspected thalassemia carriers with small mean corpuscular volume (MCV), mean corpuscular hemoglobin content (MCH) or mean corpuscular hemoglobin concentration (MCHC) but the concentration of serum ferritin within normal limits were further investigated by polymerase chain reaction (PCR) and flow through hybridization gene chip to detect common mutations of α-globin and β-globin genes using Thalassemia Geno Array Diagnostic Kit. The prevalence and genetic mutation spectrum of thalassemia among breast cancer patients were analyzed.

**Results:** Four hundred and eighty-nine suspected thalassemia carriers were detected by complete blood cell counts and serum ferritin analysis among 1887 breast cancer patients. One hundred and seven cases (5.7%) were identified as carriers of thalassemia, of which 55 cases (51.4%) were α-thalassemia, 50 cases (46.7%) were β-thalassemia, and 2 cases (1.9%) were co-inheritance of α-thalassemia and β-thalassemia simultaneously. In α-thalassemia, the most prevalent genotype is -^SEA^/αα; as for β-thalassemia, β^IVS−II−654^/β is the most common genotype. The degree of anemia is more severe in β-thalassemia than in α-thalassemia.

**Conclusion:** This is the first comprehensive molecular epidemiological investigation on thalassemia among breast cancer patients. Our data indicated that thalassemia was not uncommon in breast cancer patients. The physicians should have the knowledge to avoid misdiagnosis as iron deficiency anemia.

## Introduction

Thalassemia is one of the most frequently diagnosed inherited monogenic autosomal recessive disease caused by mutations in α-globin or β-globin gene clusters, leading to inadequate or complete loss of the corresponding globin chain synthesis and the imbalanced ratio between α-chain and β-chain in hemoglobin, which was characterized by microcytic hypochromic anemia and showed a wide spectrum of clinical severity from severe anemia to minor or none anemia (Chui et al., 2006; Shafique et al., 2021). Thalassemia can be classified into 2 major types, α-thalassemia and β-thalassemia. Of which, α-thalassemia mainly occurs at the end of the short arm of chromosome 16 α-globin gene cluster, containing α-globin gene deletion or point mutations; β-thalassemia mainly occurs in the short arm of chromosome 11 caused by point mutation within the gene (Muncie and Campbell, 2009; Brancaleoni et al., 2016). The clinical classification of thalassemia includes thalassemia minor, thalassemia intermediate (TI) and thalassemia major (TM) based on their severity of anemia (Viprakasit and Ekwattanakit, 2018). No effective treatment is available for thalassemia patients until now. Severe thalassemia patients need lifelong term blood transfusions to maintain lives, while mild or minor thalassemia has no obvious symptoms, which is usually found during pregnancy examination or annually physical examination. Thalassemia is geographically selective and widely distributed in the Mediterranean countries, Indian subcontinent, the Middle East, North Africa, Southeast Asia and other tropical and subtropical regions, including south China (Viprakasit and Ekwattanakit, 2018). The prevalence of thalassemia also varied significantly in different regions of China. Previous reports suggest a higher frequency of thalassemia in the population of southern China, particularly in the provinces of Guangdong, Guangxi, Yunnan, Fujian and so on (Zhuang et al., 2021). As patients with thalassemia under proper treatment live longer than before, it is not uncommon for thalassemia patients to develop cancer. Some researchers even believe that patients with thalassemia are more likely to develop cancer because of high levels of oxygen free radicals and iron load. However, anemia and other clinical manifestations are often misdiagnosed as iron deficiency anemia (IDA) or cancer related symptoms, which may ignore the diagnosis of thalassemia (Ansari et al., 2013; Hodroj et al., 2019). It is well-known that screening and genetic counseling during early stage pregnant are the most effective ways to prevent newborn with thalassemia, especially severe thalassemia (Jaing et al., 2021). Since people in different geographical regions having completely different mutation spectrum of globin genes, it is vital to fully understand the molecular epidemiological characteristics of incidence rate and distribution of thalassemia in order to formulate appropriate prevention strategies in areas with high incidence of thalassemia. There are quite few reports on the occurrence of malignancies among patients with thalassemia worldwide recently (Poggi et al., 2011; Haghpanah et al., 2020). Breast cancer is one of the most common diagnosed cancers and the primary causes of cancer-specific death among women. The year crude and age adjusted rate of breast cancer incidence were 45/100,000 and 30/100,000, respectively, which was ranking the first in the incidence of cancers in women in China (Lei et al., 2021). The incidence per year remained increasing both in urban areas and in rural areas. Currently, breast cancer patients are managed under multidisciplinary treatment with an approach comprising surgery, chemotherapy, radiation therapy and/or endocrine therapy. The Third Hospital of Nanchang is the largest breast cancer center in Jiangxi Province. The number of annually newly diagnosed breast cancer is over 2000 in our center, accounting nearly one-third of the new cases in Jiangxi province. Annually, about 1000 subjects received radiotherapy in the department of radiation oncology. Quite few studies have reported the occurrence of breast cancer among patients with thalassemia worldwide. After an extensive review of the literatures in PUBMED database, we found a case report on breast cancer in thalassemic patient (Picardo et al., 2015). However, neither the incidence nor the genetic mutation spectrum of thalassemia has been reported yet among breast cancer patients all over the world.

In the present study, we investigated the genetic mutation profiles of thalassemia among breast cancer patients treated in the department of radiation oncology by polymerase chain reaction (PCR) and flow through hybridization gene chip to detect common mutations of α-globin and β-globin genes using Thalassemia Geno Array Diagnostic Kit (Guangdong Hybribio Biotech Co., Ltd., Yaneng BioSciences, Zeesan Biotech). The purpose of the study is to provide valuable information for medical staff to prevent misdiagnosing thalassemia as IDA and making wrong treatment decision.

## Materials and methods

### Study subjects

A total of 1,887 subjects were enrolled during 1 April 2020 and 30 March 2022, who received radiotherapy in the department of radiation oncology, the Third Hospital of Nanchang. The age of these subjects ranged from 20 to 91 years old, and the male-to-female ratio was 1: 628. The clinicopathological characterisics including age, menopausal status, histological type, grade, TNM stage, estrogen receptor (ER) status, progesterone receptor (PgR) status, HER2 (human epidermal growth factor receptor-2) status, Ki-67 index, molecular subtypes of the patients were listed in Table 1. Written informed consents were obtained from all of the subjects. This study was approved by the Institutional Medical and Ethics Committee of the Third Hospital of Nanchang.

**TABLE 1:** The clinicopathological characteristics of the breast cancer patients enrolled in this study.

	Number	%
Age		
<35	129	6.8
35–55	1181	62.6
≥55	577	30.6
menopausal status		
Premenopausal	1219	64.6
Postmenopausal	668	35.4
histological type		
DCIS	87	4.6
IDC	1345	71.3
others	455	24.1
histological grade (IDC)		
1	39	2.9
2	674	50.1
3	632	47.0
TNM stage		
0	87	4.6
Ⅰ	227	12.0
Ⅱ	735	39.0
Ⅲ	618	32.8
Ⅳ	220	11.7
ER status		
−	582	30.8
+	1305	69.2
PgR status		
−	801	42.4
+	1086	57.6
HER2 status		
0	171	9.0
1+	348	18.5
2+	946	50.1
3+	422	22.4
Ki-67 index		
<15%	509	27.0
≥15%	1378	73.0
Molecular subtypes		
luminal A	390	20.7
luminal B	963	51.0
HER2 positive	301	15.9
TNBC	234	12.4
Surgery		
BCS	625	33.1
MRM	1255	66.5
No surgery	7	0.4

ER, estrogen receptor; PgR, progesterone receptor; HER2, human epidermal growth factor receptor-2; TNBC, triple negative breast cancer; BCS, breast conserving surgery; MRM, modified radical mastectomy.

### Hematological analysis and serum ferritin test

Approximately 3 ml of anticoagulated peripheral blood of each subject was collected into EDTA-containing vacutainers used for blood routing test. We performed complete blood cell counting on all of the subjects according to standard laboratory procedures using an automated hematology cell counter followed the standard operating procedures provided by Sysmex XN350 blood analyzer (Sysmex XN350; Sysmex Co., Ltd., Kobe, Japan). Patients with mean corpuscular volume (MCV), mean corpuscular hemoglobin content (MCH) and/or mean corpuscular hemoglobin concentration (MCHC) below lower limits of the normal threshold were further analyzed by serum ferritin test. The serum ferritin test was performed with immunochromatographic result interpretation recorder (NS7001, Tianjin, China). Patients with serum ferritin <20 ng/ml (lower limits of the normal threshold) were considered IDA and received iron supplementation. Patients with serum ferritin >20 ng/ml (lower limits of the normal threshold) and any of MCV <82 fl, MCH <27 pg and/or an MCHC <320 g/L regarded as suspected thalassemia carriers were subjected to further thalassemia genetic testing.

### Blood DNA extraction

We collected a further 2 ml of peripheral blood from every patient with abnormal small corpuscular volume red blood cells but normal concentration of serum ferritin for the molecular analysis of common α-thalassemia and β-thalassemia. Automatic blood DNA extraction kits (Guangdong Hybribio Biotech Co., Ltd., Yaneng BioSciences, Zeesan Biotech) was used to extract the genomic DNA of the subjects according to the manufacturer’s protocol. The DNA concentration and purity were determined by UV spectrophotometer (Thermo Fisher Scientific, Wilmington, DE, United States) at the wavelength of 260 nm.

### Polymerase chain reaction and flow-through hybridization and gene chip

**FIGURE 1:**
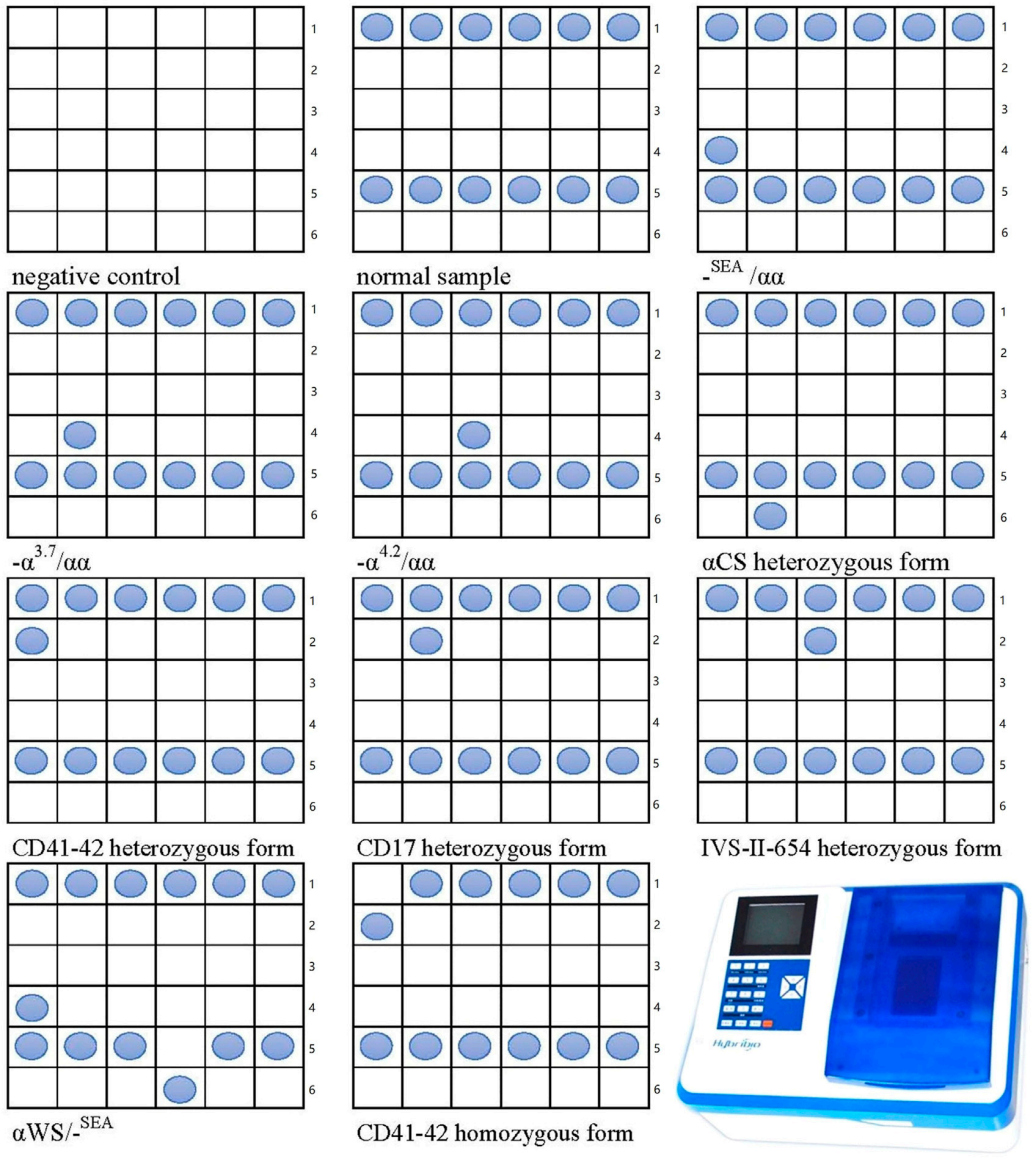
The Nucleotide template in each cell and the establishment of the genotyping of thalassemia using flow-through hybridization and gene chip. Line 1:41–42N, 17N, 654N, 71–72N, −28N, βEN; line 2: 41–42M, 17M, 654M, 71–72M, -28M, βEM; line 3: 43M, 14–15M, −30M, −32M, −29M, CapM; line 4: SEA, α3.7, α4.2, 31M, IVSⅠ-5M, IntM; line 5: NP, CSN, QSN, WSN, IVSⅠ-1N, 27–28N; line 6: blank, CSM, QSM, WSM, IVSⅠ-1M. N, normal; M, mutation.

Blood DNA was used to amplify α-thalassemia and β-thalassemia related genes with the PCR amplification Mix (Guangdong Hybribio Biotech Co., Ltd., Yaneng BioSciences, Zeesan Biotech), as previously described (Peng et al., 2021). Briefly, 5 μl DNA extraction was mixed with 45 μl reaction mixture system containing 100 pmol of α-globin or β-globin primers (Hybribio Biotechnology PCR Kit). Blood DNA was amplified in an Applied Biosystems Automated Thermal Cycler (Thermo Fisher Scientific Inc.), the amplification conditions were 95°C for 15 min, followed by denaturation for 50 s at 97°C, annealing for 60 s at 60°C, and extension at 72°C for 120 s for a total of 35 cycles. Amplification was followed by a 19 min terminal extension step at 72°C, then the products were hold on ice for further flow-through hybridization. Genotypes of thalassemia were done by flow-through hybridization and gene chip (Guangdong Hybribio Biotech Co., Ltd., Yaneng BioSciences, Zeesan Biotech). The chip could identify three common deletional α-thalassemia mutations (-SEA, -α^3.7^, -α^4.2^) and three common non-deletional α-thalassemia mutations (αCS, αQS, αWS) for α-thalassemia. The 17 common β-thalassemia mutations detected were as follows: CD41-42, IVS-II-654, –28, CD17, CD71-72, βE, –29, CD43, CD31, –32, CD14-15, CD27-28, IVS I-1, IVS I-5, Int, Cap, –30. The kit of the gene chip was approved by Chinese State Food and Drug Administration. All experimental operations were performed strictly following the manufacturers’ protocols (ADICON Clinical laboratories, Inc., Nanchang, China). The final results were detected and read by colorimetric change on the chip under direct visualization (Figure 1).

### Statistical analysis

The statistical analysis was conducted using the SPSS version 22. Results were expressed as mean ± SD values, and differences among groups were compared for each hematological index using independent sample Student’s t test or chi-square test to compare the detection rates between the groups. A *p*-value < 0.05 was considered statistically significant.

## Results

### The manifestation of microcytic hypochromia among patients with breast cancer

Five hundred and sixty-three cases with microcytic hypochromia were detected among 1887 breast cancer patients. The definition of microcytic hypochromia is mean corpuscular volume (MCV) values below 80 fl, mean corpuscular hemoglobin (MCH) values below 27 pg, according to the lower limits of the normal threshold in our central lab. Seventy-four (13.1%) of the patients with microcytic hypochromia would be due to iron deficiency as serum ferritin level below 20 ng/ml, the lower limits of the normal threshold in our central lab. The rest 489 suspected thalassemia carriers underwent further thalassemia genetic testing.

### Prevalence and mutation spectrum of thalassemia

**FIGURE 2:**
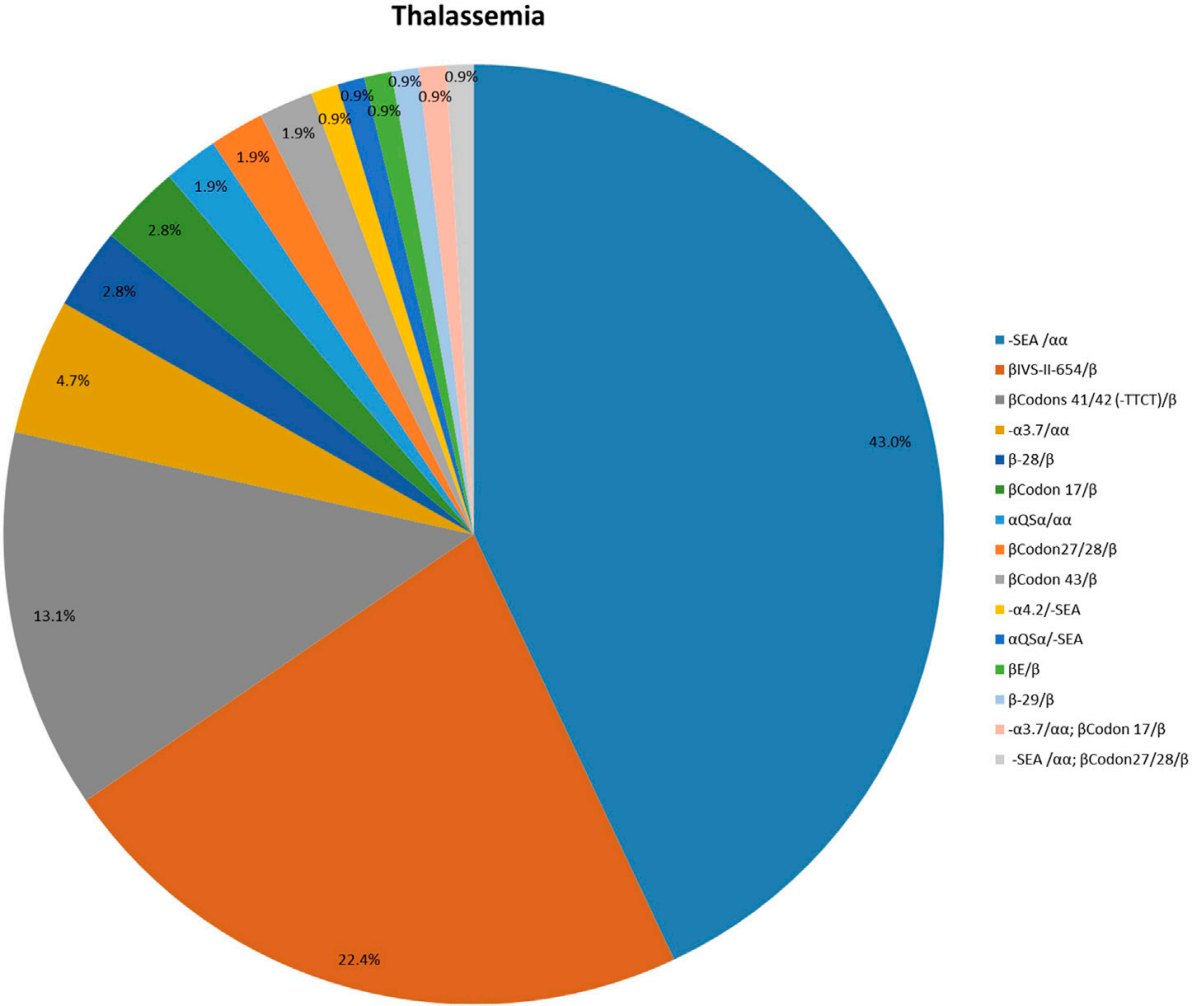
The distribution of molecular genetics spectrum of thalassemia among breast cancer patients in Jiangxi province. The -^SEA^/αα, β^IVS−II−654^/β, β^Codons 41/42^/β, -α^3.7^/αα, β^−28^/β and β^Codon17^/β were the top six common genotypes with frequencies of 43.0%, 22.4%, 13.1%, 4.7%, 2.8%, and 2.8%, respectively.

**FIGURE 3:**
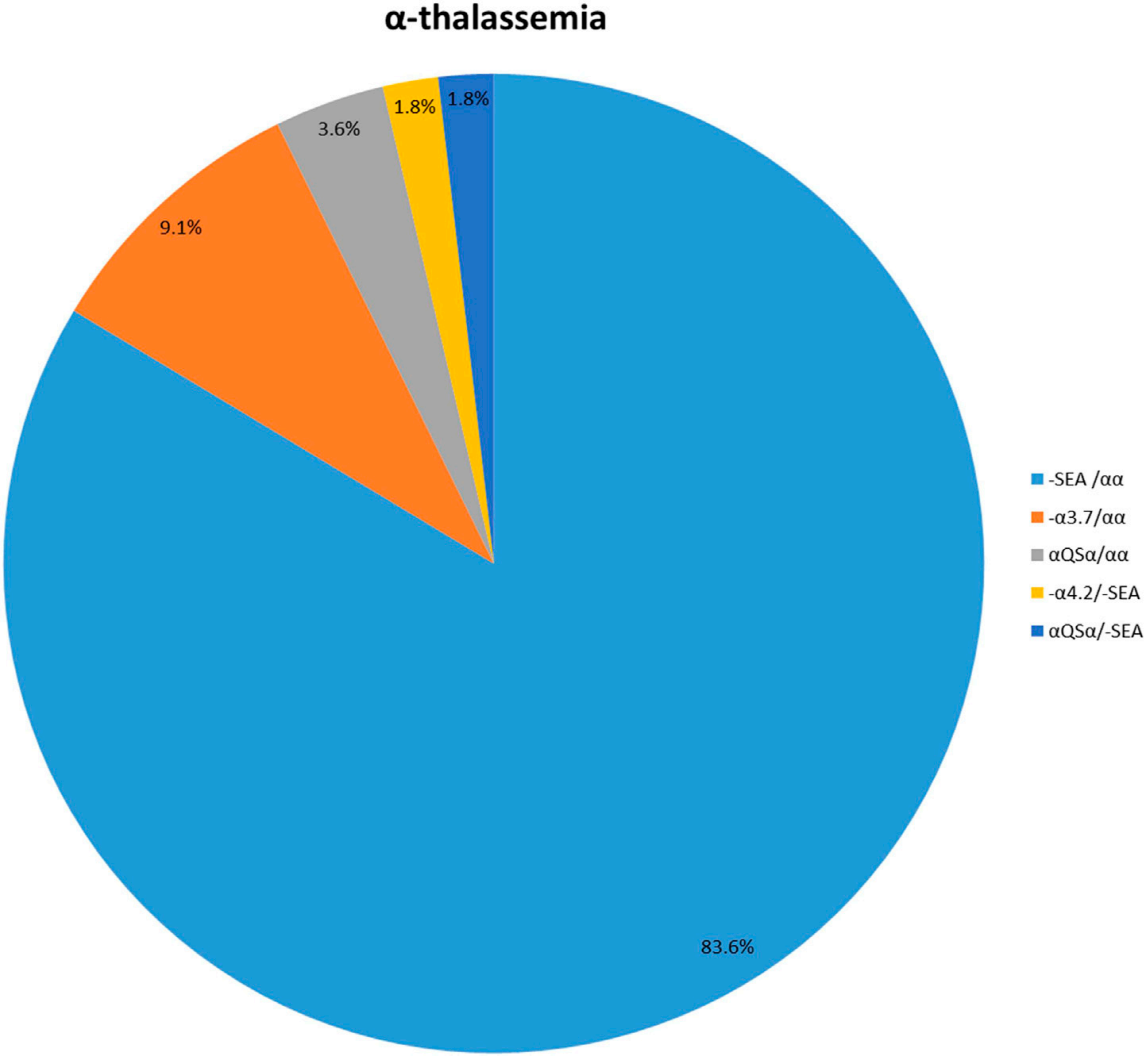
The prevalence of molecular genetics spectrum among α-thalassemia breast cancer patients in Jiangxi province. The most prevalent genotype of α-thalassemia was -^SEA^/αα, accounting for 83.6% of all α-thalassemia genotypes.

**FIGURE 4:**
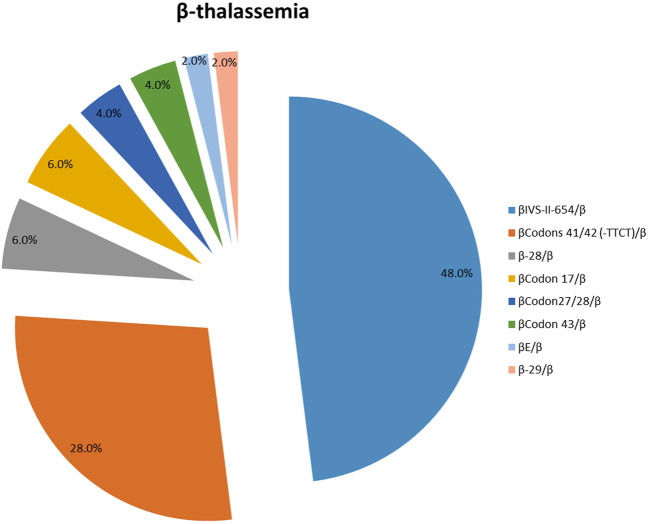
The prevalence of molecular genetics spectrum among β-thalassemia breast cancer patients in Jiangxi province. β^IVS−II−654^/β was the most common genotype, accounting for 48% of all β-thalassemia genotypes.

One hundred and seven patients were identified as thalassemia carriers, of which, 55 cases were α-thalassemia, 50 cases were β-thalassemia, and 2 cases were co-inheritance of α-thalassemia and β-thalassemia simultaneously. The overall prevalence rate of thalassemia was 5.7% among breast cancer patients in our breast cancer institute. The top six common genotypes were -^SEA^/αα, β^IVS−II−654^/β, β^Codons 41/42^/β, -α^3.7^/αα, β^−28^/β and β^Codon17^/β with frequencies of 43.0%, 22.4%, 13.1%, 4.7%, 2.8%, and 2.8%, respectively (Figure 2). The prevalence rate of α-thalassemia was slightly higher than that of β-thalassemia, and the ratio was 51.4% vs. 46.7%. What’s more, we found two carriers with both α-thalassemia and β-thalassemia, and the rate was 1.9%. Consistent with previous reports, the most prevalent genotype is -^SEA^/αα in α-thalassemia, accounting for 83.6% of all α-thalassemia genotypes (Figure 3) (Lai et al., 2017; He et al., 2021; Peng et al., 2021; Zhuang et al., 2021). As for β-thalassemia, β^IVS−II−654^/β is the most common genotype, accounting for 48% of all β-thalassemia genotypes (Figure 4).

We further explored the prevalence and mutation spectrum of thalassemia among different molecular subtypes of breast cancer. However, we found no significant difference in the prevalence and mutation spectrum of thalassemia among luminal A, luminal B, HER2 positive and triple negative breast cancer.

### Comparison of the hematological parameters among the different genotypes of thalassemia

There are quite few reports available on the hematological parameters characterization of patients with different genotypes of thalassemia. To further analyze the hematological phenotype among different genotypes of thalassemia, we performed a comparison of the hematological parameters. As Table 2 illustrating, there were significant differences of hematological parameters including Hb, MCV and MCH between α-thalassemia and β-thalassemia, while no difference in RBC count and MCHC. The percentage of clinically silent thalassemia (Hb > 120 g/L) in α-thalassemia and β-thalassemia was 21.6% and 8%, respectively. While the rate of patients with moderate degree of anemia in α-thalassemia and β-thalassemia was 2% and 12% respectively. What’s more, we detected one patient with severe anemia in β-thalassemia. No patients with extremely severe anemia was found in this population studied (Table 3).

**TABLE 2:** The differences of hematological parameters between α-thalassemia and β-thalassemia.

	α-thalassemia mean ± SD	β-thalassemia mean ± SD	*p* value
RBC (10^12^/L)	5.27 ± 0.65	5.16 ± 0.77	0.45
Hb (g/L)	112.35 ± 12.21	102.66 ± 13.85	<0.01
MCV (FL)	68.18 ± 7.71	63.05 ± 9.09	<0.01
MCH (pg)	21.57 ± 2.71	20.28 ± 2.33	0.012
MCHC (g/L)	315.41 ± 15.44	316.54 ± 11.78	0.68

RBC, red blood cell; MCV, mean corpuscular volume; MCH, mean corpuscular hemoglobin content; MCHC, mean corpuscular hemoglobin concentration.

**TABLE 3:** The degrees of anemia between α-thalassemia and β-thalassemia.

Hb (g/L)	α-thalassemia	β-thalassemia (%)	*p* value
silent (>120)	21.6%	8	
mild (90–120)	76.5%	78	
Moderate (60–90)	2%	12	
severe (30–60)	none	2	

The percentage of clinically silent thalassemia (Hb > 120 g/L) in α-thalassemia and β-thalassemia was 21.6% and 8%, while the rate of patients with moderate degree of anemia in α-thalassemia and β-thalassemia was 2% and 12% respectively. Chi-square test was applied to compare the degrees of anemia between α-thalassemia and β-thalassemia, *p* < 0.05.

## Discussion

Thalassemia is an inherited autosomal recessive disorder with the traits of microcytic hypochromic anemia. However, the clinical manifestation varied significantly, from almost normal blood test to a lethal hemolytic anemia. It is reported that the incidence of thalassemia was very high in southern China population. Lin reported that a higher frequency (9.49%) of thalassemia in Ganzhou (the south of Jiangxi province), whereas the frequency was 3.90% in Xinyu (the middle of Jiangxi province) and 2.63% in Nanchang (the north of Jiangxi province, also the capital of the province) (Lin et al., 2014).

Over the past three decades, there were several reports on the malignancies complicated with thalassemia. Some scholars even believed that patients with thalassemia would be more likely to suffer from cancer. E. Picardo et al. first reported a unique patient of breast cancer with thalassemia intermediate receiving regular red blood cell transfusions since she was two years old and began desferrioxamine after 10 transfusions (Picardo et al., 2015). The occurrence of breast cancer in patients with thalassemia may not be a pure coincidence. Some recently researches showed that there are specific mammogram patterns among thalassemic patients, nearly one-third of the patients had high density breast in mammograms, which indicated the risk of breast cancer among thalassemic patients (Rund and Rachmilewitz, 2005).

As the prevalence of thalassemia is relatively high in our province, the patients with microcytic hypochromia in complete blood cell counts receiving radiotherapy for breast cancer are not uncommon. Consequently, we conducted this study to see the distribution of thalassemia in breast cancer patients. To our knowledge, this is the first study performed to reveal the prevalence and molecular characteristics of thalassemia in the patients with breast cancer. Consistent with common population, -^SEA^/αα was also the most common thalassemia mutation in patients with breast cancer, while β^IVS−II−654^/β was the most frequent mutation in β-thalassemia.

As the largest breast cancer center in Jiangxi province, the Third Hospital of Nanchang treated about one-third of breast cancer patients in Jiangxi province. The prevalence and mutation spectrum of thalassemia among breast cancer patients received radiotherapy in the department of radiation oncology could reflect the whole situation of Jiangxi Province. As far as I am concerned, this is the first large scaled epidemiological investigation and spectrum analysis on the mutations and hematological characteristics of thalassemia among breast cancer patients. The findings of this study may provide reference for our medical colleagues with caution of breast cancer patients complicated with thalassemia. What’s more, the correlation of genotype and anemia manifestation in this study might help the clinicians to have rapid primary impression of thalassemia.

Different from previous reports, none of the subjects in this study received regular red blood cell transfusions for anemia. Tumor hypoxia has been intensively reported to be associated with decreased sensitivity to radiotherapy, previously (Wiechec et al., 2022; Yan et al., 2022; Eisenbrey et al., 2018). Anemia is thought to worsen intramural hypoxia, whose presence before or during radiation treatment adversely influences tumor radiosensitivity and is independently correlated with poor locoregional disease control and overall survival (Harrison and Blackwell, 2004; Tanaka et al., 2021). Consequently, the relationship between thalassemia and prognosis of patients with breast cancer needs further study.

## Conclusion

Thalassemia was not uncommon in breast cancer patients. The physicians should have the knowledge to avoid misdiagnosing as IDA. Since anemia may cause cell hypoxia, while hypoxic cells showing low sensitivity to radiotherapy, whether the prognosis of breast cancer patients with thalassemia is poorer after radiotherapy remains to be further studied.

## Data Availability

The raw data supporting the conclusion of this article will be made available by the authors, without undue reservation.
